# Supplementary material for: Structure Prediction and Analysis of Hepatitis E Virus Non-Structural Proteins from the Replication and Transcription Machinery by AlphaFold2
Source: Viruses. 2022 Jul 14;14(7):1537. doi: 10.3390/v14071537 (PMC9316534; doi:10.3390/v14071537)

Supplementary Figure S1. pLLDT values of the HEV3 polyprotein predicted structure . pLDDT values for each residue along protein sequences are plotted. The five domains are identified in the sequence by red double arrows.

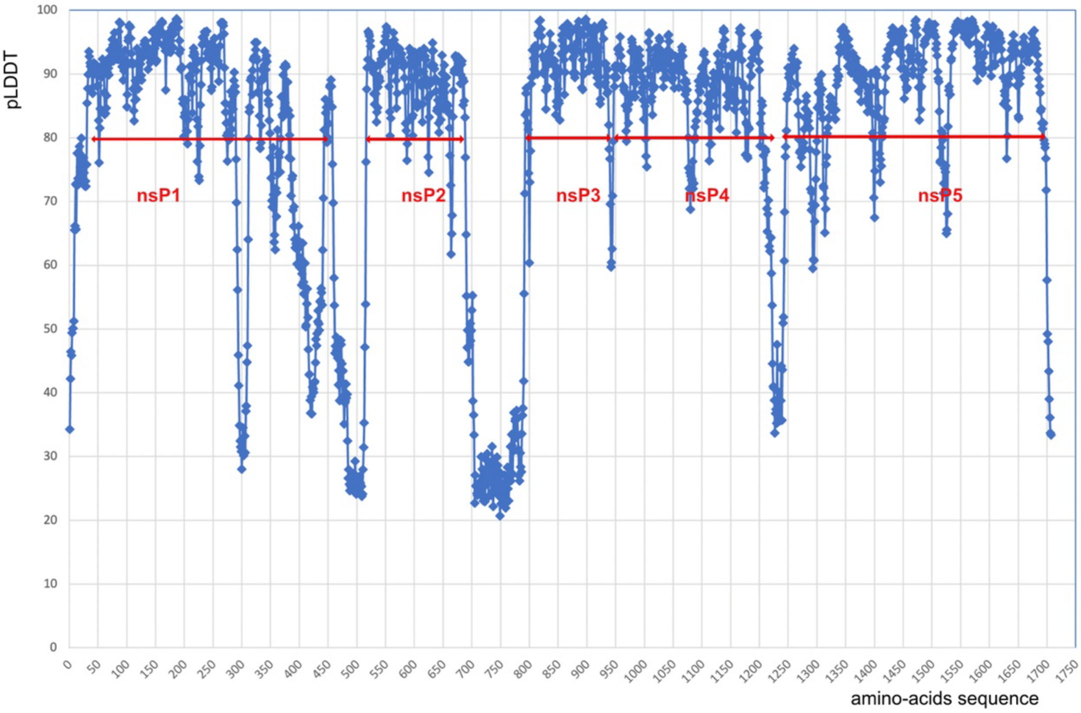

Supplementary Figure S2. Comparison of predicted and experimentally determined SARS machinery structure. A/ complex of nsP7(yellow), nsP8 (green) and nsP12 (salmon) predicted by AF2. B/ Experimental structure of nsP7(green), 2xnsP8 (salmon and yellow) and nsP12 (violet) in complex with dsRNA (green and yellow).C/ pLDDT of the predicted nsP7, nsP8 and nsP12 proteins. D/ Predicted aligned errors of the nsP7/nsP8/nsP12 predicted complex.

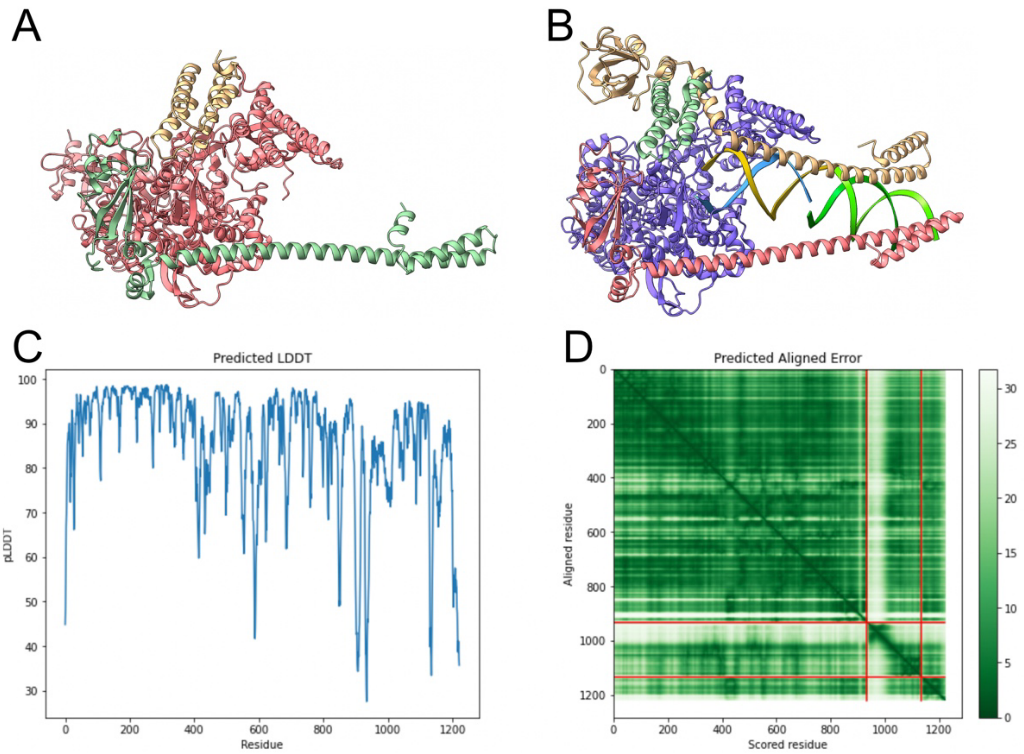

Supplement: Supplementary file 1 [file viruses-14-01537-s001.zip › supplementary figures.pdf]
